# Reproductive hormones in breast cancer bone metastasis: The role of inhibins

**DOI:** 10.1016/j.jbo.2016.03.005

**Published:** 2016-04-23

**Authors:** Caroline Wilson

**Affiliations:** Academic Unit of Clinical Oncology, Weston Park Hospital, Whitham Road, Sheffield, UK

**Keywords:** Breast cancer, Bisphosphonates, Inhibin, Activin, Follistatin

## Abstract

The spread of breast cancer cells to bone and survival in this new metastatic environment is influenced not only by the genetic signature of the cells, but also multiple host cells and soluble factors produced locally (paracrine) or from distant sites (endocrine). Disrupting this metastatic process has been evaluated in clinical trials of the bone targeted agents bisphosphonates and denosumab and have shown that these agents reduce the recurrence of breast cancer in postmenopausal women only, suggesting the efficacy of the drugs are influenced by levels of reproductive endocrine hormones. The molecular mechanism driving this differential effect has not been definitively identified, however, there is evidence that both reproductive hormones and bisphosphonates can affect similar paracrine factors and cellular components of the bone metastatic niche. This review focuses on how the ovarian endocrine hormone, inhibin, interacts with the paracrine factors activin and follistatin, abundant in the primary tumour and bone microenvironment, with subsequent effects on tumour cell survival. Inhibin also affects the cellular components of the bone microenvironment primarily the osteoblastic niche. Recent evidence has shown that bisphosphonates also alter this niche, which may represent a common mechanism by which inhibin and bisphosphonates interact to influence disease outcomes in early breast cancer. Further research is needed to fully elucidate these molecular mechanisms to enable understanding and future development of alternative bone targeted treatments with anti-tumour efficacy in premenopausal women.

## 1 Introduction

Breast cancer commonly spreads to bone in a process involving migration of tumour cells through the stroma followed by intravasation, homing to and extravasation at distant sites such as bone, and ultimately survival in this new metastatic environment. The survival of tumour cells during this process is influenced by their genetic signature and a plethora of host cells and soluble factors [1]. Disrupting the process of metastatic spread from primary breast tumour to bone was evaluated using the osteoclast inhibitors, bisphosphonates, in (neo)adjuvant clinical trials, with the hypothesis that preventing osteolysis, and release of tumour promoting growth factors from bone, may inhibit tumour cell survival. Bisphosphonates were found to improve survival only in women who were naturally or chemically postmenopausal when treatment was started [2]. The molecular mechanism for this differential effect of bisphosphonates according to menopausal status is currently unknown, but there is evidence that female hormones, such as inhibin, can interact with paracrine factors known to affect tumour cell growth in both the breast primary tumour and the bone microenvironment.

### 1.1 Menopause is associated with change in ovarian hormones affecting bone

Menopause is characterised by a decrease in ovarian oestradiol and inhibins with an increase in pituitary follicle stimulating hormone (FSH). The decline in inhibins drives the increase in bone turnover that occurs in early menopause and although inhibins are not abundantly expressed in bone, radiolabelled inhibin A administered intravenously *in vivo* accumulates rapidly in the bone marrow indicating that it can distribute to bone (reviewed in Wilson et al.) [3]. In a cross sectional study of women aged 21–85 (n=188), endocrine hormones were correlated to changes in serum markers of bone formation; bone specific alkaline phosphatase (BSAP), and bone resorption; carboxyterminal telopeptide of type I collagen (CTX). Inhibin A was shown to be the most accurate predictor of changes in bone formation and resorption being negatively correlated with levels of BSAP and CTX [4], thus declining inhibins in early menopause will lead to increased bone turnover. The primary role of inhibins is to inhibit the secretion of FSH from the anterior pituitary and thus the role of inhibins must be considered in the context of associated changes in FSH. In the cross sectional study FSH correlated with bone resorption markers (CTX) but not bone formation markers (BSAP) in perimenopausal women, and did not correlate with any bone turnover markers in pre- or postmenopausal women [4]. *In vitro*, FSH increases osteoclast differentiation [5], and *in vivo* treatment of ovariectomised 14-week old mice with an antibody to β-subumit of FSH, blocking its biological activity, prevents OVX-induced bone loss after 4 weeks of treatment. Dynamic histomorphometry showed inhibiting FSH increases all bone formation parameters and inhibits bone resorption parameters [6]. In contrast, a prospective study of changes in bone turnover in postmenopausal women (n=46) with inhibition of FSH, using GnRH agonists, showed a significant increase in CTX and TRAP5b (serum markers of bone resorption) with suppression of FSH, in addition to a significant increase in P1NP (a marker of bone formation) [7]. These data suggest FSH does not directly regulate bone resorption in postmenopausal women, however lowering FSH levels may affect bone formation either directly by affecting number or activity of the bone forming cells; osteoblasts (Ob), or indirectly due to the coupling effect of bone turnover following an increase in bone resorption.

### 1.2 Molecular interactions of endocrine and paracrine factors; implications for tumour growth

**Fig. 1:**
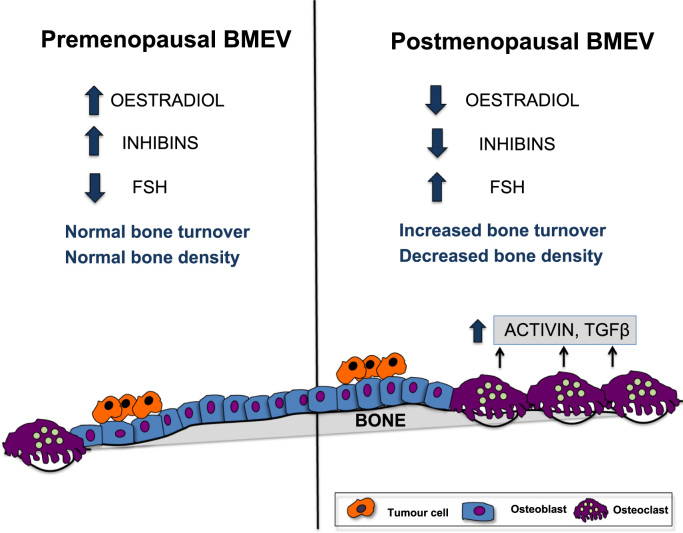
The endocrine changes in bone as a result of menopause. The menopause induces changes in both endocrine and paracrine factors in the bone microenvironment. Ovarian failure increases bone turnover due to a decline in ovarian inhibins and oestradiol. As a result of increased bone turnover osteoclastic bone resorption releases soluble factors that are stored in bone, *i.e.* activin and TGFβ. These paracrine soluble factors can influence tumour cells in the bone microenvironment (BMEV).

Inhibins do not have an identified intracellular downstream signaling pathway but bring about their effector functions by inhibiting ligand: receptor interaction of the soluble paracrine factors activin and TGFβ, abundant in both the primary tumour and bone microenvironment [8]. Activin and TGFβ each bind to their respective type II receptors, but both recruit the same type I receptor resulting in phosphorylation of the receptor associated Smads 2/3 [9]. Activin is a tumour suppressor that is bound to a single chain glycosylated peptide, follistatin, from which it must be cleaved to allow receptor ligand interaction. Thus, female hormones such as inhibin may affect cancer cell survival through modification of tumour paracrine factors (reviewed in Wilson et al. [3]). Three key *in vivo* studies [10], [11], [12] have investigated the effect of blocking the activin type IIA receptor (ActRIIA) on bone, either with inhibin A or a soluble extracellular domain of ActRIIA fused to a murine IgG2a-Fc. These studies have collectively demonstrated that blocking this receptor increases bone density in mouse models by increasing the activity and number of Ob. The subsequent effect on tumour cell survival and growth in bone remains to be established, but the bone microenvironment will differ according to menopausal status not only at a cellular level but also in terms of the soluble factors present. Activin is stored in the bone matrix and produced locally in the bone marrow during osteoclast mediated bone breakdown [13]. Bone activin levels would therefore be expected to be low in quiescent premenopausal bone due to high inhibin levels and low bone turnover, with the converse true in postmenopausal bone (Fig. 1).

### 1.3 Endocrine:paracrine influence in the breast primary tumour and interaction with bisphosphonates

Activin is secreted by breast cancer cell lines *in vitro* and inhibits proliferation [14]. In clinical breast cancer samples loss of expression of the activin type II receptor is associated with increasing tumour grade [15], confirming the tumour suppressive activity of activin in breast cancers. Breast tumour cells can impair activin signaling with evidence that follistatin, secreted by tumour cells, promotes tumour growth [16] and inhibin A promotes stomal invasion and metastasis [17].

*Hormone interaction with bisphosphonates*; the bisphosphonate zoledronic acid (ZA) has been shown to increase activin's biological activity in breast cancer cells *in vitro* and *in vivo*, enhancing its tumour suppressive effects [18]. Moreover, postmenopausal breast cancer patients receiving ZA+neo-adjuvant chemotherapy (CT) show a significant fall in serum follistatin levels compared to CT alone, thus increasing activin's bioavailability in these patients (an effect not seen in premenopausal patients) [19]. A meta-analysis of four clinical trials evaluating the addition of ZA to neo-adjuvant CT (n=553) found that postmenopausal women have improved pathological complete response rates (pCR) in primary breast tumours when treated with ZA plus CT *vs* CT alone (13.6% *vs* 7.8%, respectively) [20] indicating that low levels of endocrine hormones enhance the response of primary tumours to bisphosphonates.

### 1.4 Endocrine:paracrine influence on the homing of tumour cells to bone and interaction with bisphosphonates

Disseminated tumour cells are detectable in the bone marrow of a third of patients with early breast cancer without any clinical manifestations of bone metastasis [21]. A meta-analysis of over 4000 bone marrow aspirates from breast cancer patients without bone metastases found that premenopausal patients had a significantly higher prevalence of bone marrow disseminated tumour cells (DTCs) than postmenopausal women (32.7% *vs* 29.5%) [21], suggesting premenopausal bone (with low activin levels due to ovarian inhibin) may attract tumour cells. This is in contrast to *in vivo* data showing that lowering activin levels with a soluble activin receptor type IIA fusion protein prevents the formation of bone metastases from MDA-MB-231 cells [10], thus the role of activin and inhibin in modifying the bone microenvironment and survival of tumour cells needs defining, and may differ in the preclinical and clinical settings.

*Hormone interaction with bisphosphonates;* Clinical trials have shown that bisphosphonates decrease the number of bone marrow DTCs in marrow aspirates from breast cancer patients [22], [23], [24], [25]. Since DTCs have not developed autonomous growth, it is likely that this effect is mediated through bisphosphonate-induced changes in the bone microenvironment, rather than a direct anti-tumour effect. The influence of hormones on the ability of bisphosphonates to eliminate DTCs was not possible to assess in these clinical trials since they were not large enough to show a differential effect of bisphosphonates according to menopausal status.

### 1.5 Endocrine influence on the survival of tumour cells in bone and interaction with bisphosphonates

50% of patients with detectable DTCs will relapse during 10 years post diagnosis [21], however some patients will never develop bone metastases thus survival of tumour cells in the bone depends on a conducive bone microenvironment. A study of incidence of bone metastases in >7064 breast cancer patients showed that younger patients were more likely to develop bone metastases and in multivariate analysis the hazard ratio of developing bone metastases was highest in women who were aged under 40 years at diagnosis (HR 0.47 95%CI 0.38–0.57) [26], suggesting premenopausal bone may provide a more favorable environment for tumour cell survival and growth into clinically overt bone metastases. Standard chemotherapy agents directly target proliferating tumour cells, however, there is increasing evidence that the bone microenvironment may be a potential sanctuary for DTCs that are dormant and resistant to conventional systemic anti-cancer therapies [27]. It is possible that DTCs may localise to the haematopoetic stem cell (HSC) niche in bone and use the same stromal adhesion and environmental signals to enable them to survive. It has been shown that tumour cell lines with bone homing properties express the receptor for annexin II that Ob use to adhere to HSCs and knockdown of this receptor in prostate cells prevented bone metastasis after intacardiac injection [28].

*Hormone interaction with bisphosphonates;* Disrupting the interactions between DTCs and the bone stromal cells was evaluated in phase III randomised clinical trials of adjuvant bisphosphonates, and a large meta-analysis of these adjuvant bisphosphonate trials involving >18,000 patients showed bisphosphonates prevented breast cancer recurrence in bone (RR 0.72, 0.60–0.86; 2p=0.0002), at other distant sites (RR 0.82, 0.74–0.92; 2p=0.0003) and improved breast cancer mortality (RR 0.82, 0.73–0.93; 2p=0.002) in women who were postmenopausal when treatment started [2]. Recent data from the ABCSG-18 trial has shown that adjuvant treatment with the RANK ligand inhibitor denosumab, which prevents osteoclast activation, reduces the risk of disease recurrence in postmenopausal patients with early stage hormone receptor positive breast cancer [29], suggesting that osteoclast inhibition by an alternative molecular mechanism to bisphosphonates also improves outcomes in patients with low levels of female hormones.

## 2 Discussion and outstanding questions

Reproductive endocrine hormones such as inhibin affect breast cancer cell survival in the primary tumour and affect tumour homing and survival in the bone microenvironment. The molecular mechanism driving this effect of hormones in bone is likely to be multifactorial by modification of both paracrine factors and the cellular components of the bone metastatic niche. If the Ob niche is key to the maintenance of dormancy and survival of tumour cells then factors that affect the size of this niche can potentially determine outcomes for breast cancer cells in this environment. There is evidence from *in vivo* models that expanding the Ob niche with the use of parathyroid hormone, increased the number of DTCs in bone from sub-cutaneous prostate tumours [30]. Zoledronic acid has also been shown to have effects on the Ob niche and a recent *in vivo* study evaluating the effects of a single dose of zoledronic acid (100 μg/kg) showed that the drug significantly reduced Ob number which influenced where intracardiac injected MDA-MB-231 breast cancer cells home to, with a preference demonstrated for Ob rich areas [31]. Both inhibin and bisphosphonates differentially affect paracrine factors and cellular components of the bone metastatic niche, in particular the Ob niche, with potentially differential effects on the survival of DTCs in bone. The specific molecular mechanisms driving this need further elucidation to allow understanding and development of alternative bone targeted treatments that are mechanistically different to the osteoclast inhibitors and have anti-tumour efficacy in premenopausal women.

### 2.1 Outstanding questions

• Which female hormone(s) affect the direct anti-tumour efficacy of bisphosphonates in primary tumours and the indirect anti-tumour efficacy of bisphosphonates in bone, in particular what are the roles of activin and inhibin?
• What are the cellular/molecular drivers of tumour growth in the pre- and postmenopausal bone microenvironments?
• Is the differential effect of menopause on the anti-tumour efficacy of osteoclast inhibitors specific to breast cancer patients only or does this apply to other tumours such as lung cancer?
